# Postural Stability of Adolescents with Late Cochlear Implantation and Hearing Aids: A Non-Randomized Trial

**DOI:** 10.3390/audiolres14040048

**Published:** 2024-06-25

**Authors:** Anna Zwierzchowska, Eliza Gaweł, Agata Krużyńska, Kajetan J. Słomka, Aleksandra Żebrowska, Grzegorz Juras

**Affiliations:** 1 Institute of Sport Sciences, The Jerzy Kukuczka Academy of Physical Education, 40-065 Katowice, Poland; a.zwierzchowska@awf.katowice.pl (A.Z.); k.slomka@awf.katowice.pl (K.J.S.); a.zebrowska@awf.katowice.pl (A.Ż.); g.juras@awf.katowice.pl (G.J.); 2 The School and Preschool Complex for Deaf and Hard of Hearing Children, 40-126 Katowice, Poland; akruz@interia.pl

**Keywords:** hearing loss, deafness, cochlear implant, balance, vestibular disfunction

## Abstract

**Background:** The aim of this study was to assess the neuromuscular control of adolescents with late unilateral cochlear implantation and compare them to adolescents with hearing aids (HAs) while performing a balance task on a platform with the conditions of an activated hearing device (cochlear implant (CI)/HAs) with eyes opened/closed (EO/EC). **Methods:** Forty-eight adolescents with hearing loss participated in the study and were divided into SG (unilateral CI and HA) and CG (bilateral HA). The evaluation of the postural stability was performed with a force plate during two repeating testing trials with EO/EC. **Results:** SG was characterized by greater values of vCOP compared to CG (EO), while, in CG, greater values of vCOP were noted in the second trial. The type of hearing device was found to be related to the values of area (EO) (*p* < 0.001), which were always greater in SG, regardless of the visual perception. **Conclusions:** Late unilateral CI may impact the activation of different models of the auditory compensatory mechanism than HA, which is related to neuromuscular control. The values of vCOP can be predicted by age in late-CI individuals. Visual perception seems not to be related to the values of the area, which can be impacted both by CI and HA.

## 1. Introduction

The proper morphofunctional and structural development in all stages of human ontogenesis is known to be intrinsically related to the reception of sound [1]. Furthermore, motor development is not only focused on controlling the activity of the muscle fibers but also on the anticipation and adaptation of the body’s structures to maintain postural stability [2], defined as a complex process that requires the active involvement of multiple human systems. These systems include (a) the sensory system, (b) the central nervous system (to process the stimulus and plan and coordinate kinesthetic tasks), and (c) the vestibular system [2]. Information from the external environment, e.g., about the location of the body segments relative to external objects, is gathered both by the visual and somatosensory systems, while the vestibular system is focused on providing information about the internal frame of reference, e.g., the orientation of the body segments in the space [2].

The available scientific literature indicates that the compensatory mechanisms that are activated due to hearing loss impact the efficiency of both visual and proprioceptive sensitivity and, as a result, the efficiency of the whole musculoskeletal and central nervous systems, including the speed of forming the neural connections between vestibular organs [3,4]. Therefore, in the past few years, cochlear implantation has become a popular solution for minimizing the incidence and influencing the compensatory mechanisms related to hearing loss [5]. It should also be noted that cochlear implantation is known to be the only opportunity for the rehabilitation of auditory communication in the case of severe and profound hearing loss [6]. Moreover, during the last two centuries, cochlear implants (CIs) have evolved from a prototype of electronic stimulation that was developed in 1790 [7] to a modern hearing device that samples the acoustic environment, processes the obtained signal into the frequency bands, and compresses the amplitude to an adequate electrically useable range [6]. That enables stimulating the neural elements to reproduce both frequency and amplitude that analyze the capacity of the cochlea [6]. 

However, due to the sensitive nature of the surgical interventions during implantation, it is difficult to rule out the possibility of complications in neuromotor control, including temporary or permanent postural disorders. Furthermore, the available scientific research has yielded partially inconsistent findings on the abovementioned research issue. For instance, some studies suggested that an early age at cochlear implantation surgery can significantly impact the level of a child’s holistic development (verbal and motor abilities and social behavior) [8], while others were concerned with the age of the patients eligible for cochlear implantation surgery [9]. Cochlear implantation has been the subject of previous investigations [10,11], yet the data on the age of implantation and postural control remain limited. Some studies suggest that the postural control system can be improved after cochlear implantation surgery [12,13,14] by adapting to the habitual posture, but, at the same time, they indicate the need for further and deeper analysis. Even though the possible effects of early cochlear implantation surgery have been examined, there is still a lack of research assessing the neuromuscular control in late unilateral cochlear implantation adolescents.

Given the above and the gap in the scientific literature, the aim of this study was to assess the neuromuscular control of adolescents with late unilateral cochlear implantation and compare them to adolescents with hearing aids (HAs) while performing a balance task on a platform (habitual position) with the conditions of an activated hearing device (cochlear implant (CI)/HA) with eyes opened/closed. It was assumed that the etiology of hearing loss and time of CI surgery are the main factors impacting the neuromotor control of the body posture. 

## 2. Materials and Methods

### 2.1. Study Design

The research was conducted with the use of a non-randomized study protocol in order to assess the neuromuscular control during postural stability performance with different visual conditions. 

### 2.2. Participants

The present study examined 48 (female = 24, male = 24; age = 16.9 ± 1.9) adolescent students with hearing loss from the four special schools and special education centers in Poland. The inclusion criteria were as follows: (1) severe bilateral sensorineural hearing loss, (2) students with a unilateral cochlear implant and unilateral hearing aid in the opposite ear or students with bilateral hearing aid (3) at least above 3 years old at the time of CI surgery (4) at least 2 years after implantation surgery (only CI students), (5) males and females aged 14 to 20 years, (6) no health condition except the hearing impairment, (7) satisfactory self-reported health status, (8) congenital or acquired impairment, and (9) free from musculoskeletal or neuromuscular disorders. The exclusion criteria were as follows: (1) children or adults with hearing loss, (2) an injury reported in the last two months, (3) no parental consent for participation in the study, (4) withdrawal from the study, and (5) multiple impairments/dysfunctions. 

Study participants were divided into two groups, i.e., a study group (SG) of unilateral CI students with HA in the opposite ear (n = 27, female = 13, male = 14) and a control group (CG) of students with bilateral HA (n = 21, female = 11, male = 10). Furthermore, 33.3% of the students from the SG underwent CI surgery before the age of 6 years, while 66.6% of them underwent the surgery after that age. Moreover, the majority of the study participants, i.e., 66.6%, underwent HA before the age of 1 year, while 33.3% of them started using HA after. Table 1 provides detailed characteristics of the study participants. 

The measurements were carried out in the laboratories of the Institutes of Sport Sciences at the Academy of Physical Education in Katowice, Poland, and in specially adapted rooms in the special schools and special education centers to which study participants attended. Informed consent was obtained from all the participants and legal guardians of the participants who were below 16 years of age. Moreover, study participants were allowed to withdraw from the experiment at any time and were informed about the benefits and potential risks of the study, and they were instructed to maintain their normal dietary and sleeping habits for 24 h before the examination. The research protocol was approved by the Bioethics Committee for Scientific Research at the Academy of Physical Education in Katowice, Poland (No. 9/2012), and met the ethical standards of the Declaration of Helsinki 2013. 

### 2.3. Procedures

Regardless of the place of examinations (certified Laboratories of the Institute of Sport Sciences, Academy of Physical Education in Katowice, Poland/specially adapted rooms in the special schools/special education centers), study participants arrived in the morning (8–11 a.m.). Firstly, an interview was conducted with the patients and their parents/legal guardians to collect information about CI/HA and the participant’s health status. Next, the anthropometric measurements and postural stability testing were performed. All measurements were taken in the same temperature conditions, using the same research tools, order, and number of measurements, including postural stability tests. 

### 2.4. Anthropometric Measurements

All anthropometric measurements were performed using a research protocol that included the assessment of the qualities and indices of body build and posture. Body height (BH) was evaluated with a wall-mounted stadiometer with a tape measure with an accuracy of 1 cm. Body mass (BM) was evaluated based on body weight (TANITA TBF-300M) with an accuracy of 0.1 kg. Fat mass (FM), fat free mass (FFM), and total body water (TBW) were measured with TANITA BC-420 MA using the bioelectrical impedance analysis. Body mass index (BMI) was calculated from the standard formula [15]. All measurements were performed by the same researchers (A.K. and A.Z.) with expertise in anthropometric evaluations. 

### 2.5. Postural Stability Testing and Procedure

To evaluate the postural stability, a force plate (AccuGait AMTI, Watertown, MA, USA) with computer software (NetForce v. 3.05) with a sampling frequency of 50 Hz (no filtering signal) was used in the study. The measurements included all three force components (Fx, Fy, and Fz) and the moment of force in relation to the center of the platform and around all three axes (Mx, My, and Mz). The resultant of the center of feet pressure in anteroposterior (COP-Y and COP-AP) and unilateral (COP-X and COP-ML) directions was calculated based on the abovementioned variables. Moreover, the evaluation of the postural balance was performed according to the methodology proposed by Zatsiorsky et al. [16]. 

Each measurement consisted of 2 trials on the force plate to determine the velocity of the center of the foot pressure (vCOP) and the area of an ellipse (area). The vCOP is a critical measure in assessing postural stability and control. vCOP quantifies the speed at which the center of pressure shifts within the base of support, providing insights into the dynamic aspects of balance. In this study, vCOP serves as a key outcome measure, enabling us to evaluate the subtle differences in postural adjustments between the SG and CG) under various sensory conditions. By analyzing vCOP, we can detect potential deficiencies in postural control mechanisms, which is particularly relevant when assessing the impact of sensory aids such as cochlear implants or hearing aids. The inclusion of vCOP in our methodology enables a comprehensive evaluation of balance and stability, offering valuable data to understand the interplay between sensory inputs and postural control.

**Figure 1:**
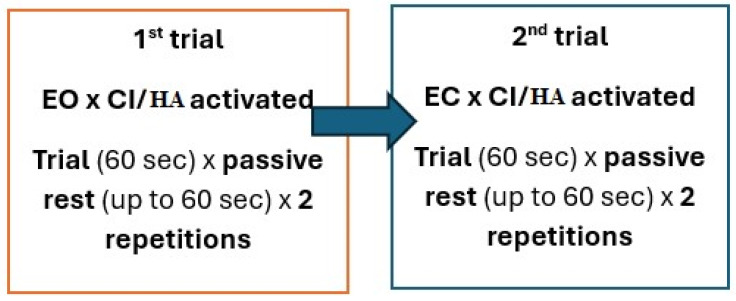
Schematic representation of the non-randomized trial design.

The trials were repeated twice with the presence of a participant’s parent/legal guardian. Moreover, all postural stability trials were supervised by the same researchers with expertise in force plate measurements and hearing impairments (K.S. and A.K.). Before the beginning of the testing, study participants were instructed (also in sign language) about the study protocol (see Figure 1) (2 trials × 2; time of each trial = 60 s) and the main testing task, i.e., to keep the most motionless position possible while standing on the force plate. After completion of each trial, the study participant descended the force plate, took a short rest (individually adjusted to the participant’s needs, up to 60 s), and entered the platform once again to begin the next trial. All breaks were measured with a hand-held timer by the same researcher (K.S.), while all measurements were recorded with the computer software (NetForce). The trial started with a verbal instruction ‘PLEASE ENTER’ and ended with a comment ‘PLEASE GET DOWN’.

The study used the following 2 trials conducted twice, both with eyes opened and eyes closed:

1. Standing in the habitual position (arms along the body) with regular loading of the feet, looking straight ahead, eyes open (EO), and cochlear implant/hearing aids activated (trial time = 60 s).
2. Standing in the habitual position (arms along the body) with regular loading of the feet, looking straight ahead, eyes closed (EC), and cochlear implant/hearing aids activated (trial time = 60 s).

Due to the difficulties in performing the whole trial in a continuous way, it was decided to use only the selected parts of the measurement in which the trial was performed correctly.

### 2.6. Statistical Analysis

All statistical analyses were performed with the STATISTICA 13.3 computer software (TIBCO Software Inc., Tulsa, OK, USA). Distributions, homogeneity, means, and standard deviations (SD) with 95% confidence intervals of the anthropometric variables and participant’s characteristics (age, age of CI, and duration of using of the hearing device) were verified using the Kolmogorov–Smirnov test. Due to the non-normal distribution between SG and CG, in order to minimize the impact of group diversity on the measurement time, an adjustment and Tukey’s post-hoc test were used. The chi-squared test of independence was performed to determine the statistically significant relationships between the categorical variables (etiology of hearing loss). The variety between vCOP (adjusted), area (adjustment), and CI/HA was verified with the Mann–Whitney U test with Bonferroni correction. A multiple regression analysis was performed in order to verify the impact of the selected clinical (age, gender, age of CI, etiology of hearing loss, and participant’s age) and somatic (foot length, BH, BM, FM, FFT, TBW, and BMI) variables on the postural stability depending on the used hearing device (CI/HA) in the conditions of EO/EC. Correlations were evaluated as trivial (0.0–0.09), small (0.10–0.29), moderate (0.30–0.49), large (0.50–0.69), very large (0.70–0.89), nearly perfect (0.90–0.99), or perfect (1.0) [17].

## 3. Results

The age of the diagnosis of the hearing impairment among the study participants with CI and HA was as follows: (1) up to 1 year old—52% (CI) and 71% (HA), (2) from 2 up to 3 years old—22% (CI) and 19% (HA), and (3) after 3 years old—7% (CI) and 10% (HA). The statistical analysis indicated statistically significant relationships (*p* < 0.05) between the number of used CIs or HAs and the time of diagnosis of hearing loss up to 1 year of age. Moreover, the chi^2^ test indicated a statistically significant higher occurrence of congenital compared to acquired hearing loss (*p* < 0.05). Simultaneously, the chi^2^ test did not indicate statistically significant differences between the type of hearing impairment (congenital/acquired) and the hearing apparatus used by the study participants (CI/HA) (*p* > 0.05).

**Figure 2:**
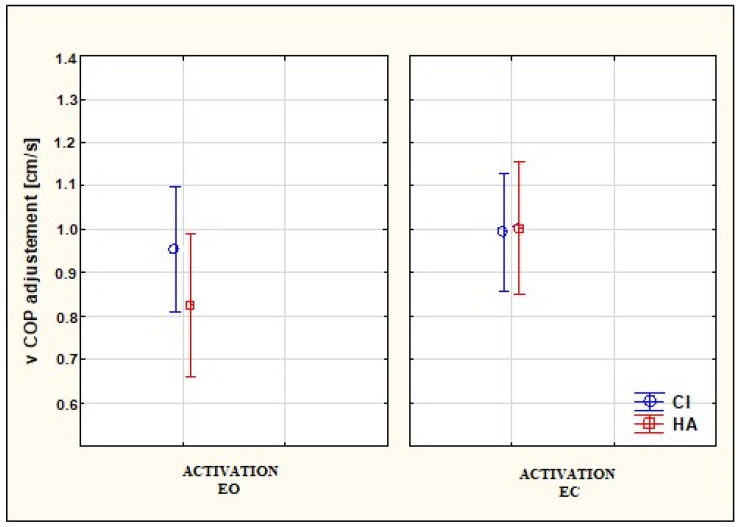
Differentiation of vCOP (velocity of the center of foot pressure, adjusted) (*p* < 0.05) in the SG (study group) and CG (control group) under the conditions of EO × CI/HA activated (eyes open with cochlear implant/hearing aid activated) and EC × CI/HA activated (eyes closed with cochlear implant/hearing aid activated). Higher vCOP values indicate greater speeds of center of pressure movement, which may suggest reduced postural stability.

The differentiation of vCOP (adjusted) in SG and CG with the condition of EO × CI/HA activated and EC × CI/HA activated is presented in Figure 2. The Mann–Whitney U test with Bonferroni correction and Tukey’s post-hoc tests did not confirm any statistically significant relationships between the type of hearing device (CI/HA) and vCOP with the condition of EO in both groups. The only statistically significant relationships were found for the changes in visual perception, i.e., EO/EC (see Figure 2). The participants with CI (SG) were characterized by greater values of vCOP compared to those with HA (CG) with the condition of EO. However, CG was found to have greater values of vCOP with the condition of EC. 

Moreover, the multiple regression analysis indicated that, in the case of students with CI, age was statistically significantly related to the values of vCOP. It was found that, along with the years of age, lower velocity of vCOP (habitual position) was observed in SG regardless of the visual perception (EO/EC), which was not observed in CG.

**Figure 3:**
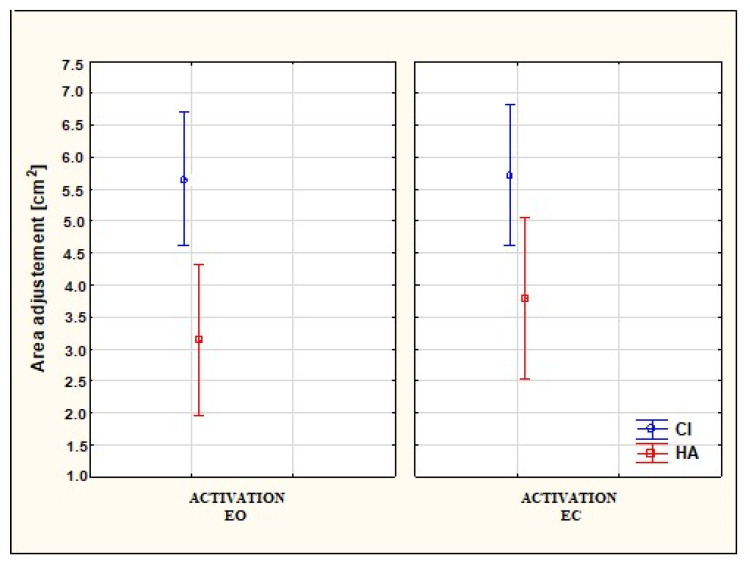
Differentiation of the area (adjusted) (*p* < 0.05) in the SG (study group) and CG (control group) under the conditions of EO × CI/HA activated (eyes open with cochlear implant/hearing aid activated) and EC × CI/HA activated (eyes closed with cochlear implant/hearing aid activated).

Figure 3 presents the results of a Mann–Whitney U test with Bonferroni correction of the area (adjusted) (*p* < 0.05) between SG and CG with the conditions of EO × CI/HA activated and EC × CI/HA activated. A statistically significant effect for the area due to the type of hearing device (CI/HA) was found in the first trial (*p* < 0.01). Moreover, no statistically significant relationships were found for the area due to the visual perception, i.e., EO/EC. Furthermore, the participants with CI always had greater area values regardless of the condition of EO/EC compared to the students with HA. 

The summary of the multiple regression for area (adjusted) with the conditions of EO/EC and hearing aid/s activated (CI/HA) according to the somatic and clinical variables in SG and CG indicated that the area did not show any statistically significant changes due to the impact of the selected clinical and somatic variables regardless of the visual perception (EO/EC) (*p* > 0.05).

## 4. Discussion

The purpose of the present study was to assess the neuromuscular control in adolescents with late unilateral CI and compare it to adolescents with HA while performing a balance task on a platform (habitual position) with the conditions of an EO/EC × activated hearing device (CI/HA). In contrast to the study hypothesis, this study revealed that both a type of hearing device (CI/HA) and visual perception are significant factors in the neuromuscular control of the body posture during standing in the habitual position. Simultaneously, age was found to impact the values of vCOP in the CI group, which partially supports our initial hypothesis. Moreover, these results show that even late unilateral CI leads to significant changes in the neuromuscular control of the body posture regardless of the visual perception. 

Our study confirmed the suggestion by An et al. [18] that the differentiation of the postural stability between CI and HA students is related to both visual and somatosensory compensation. The significant increase in the values of vCOP with the condition of EC in the HA group (CG) that was not observed in the CI group (CG) may be related to different somatosensory compensation models that are intrinsically used in both groups. As indicated by An et al. [18], the postural control in individuals with hearing impairments seems to be more highly dependent on visual than somatosensory input. This thesis indicates that a visual stimulus may be the predominant variable in the neuromuscular control of the study participants. However, due to the differentiation in the auditory devices used in both groups, such an increase in vCOP in the HA during the trial with EC indicates the dominant impact of the kinesthetic–tactual–auricular compensatory model. At the same time, the significantly higher values of vCOP in SG that were observed during the first trial and a minor increase in its values in the second suggest that, in the case of CI individuals, the opposite model of compensation is predominant, i.e., the kinesthetic–auricular–tactual model. This seems to be confirmed by the studies of Louza et al. [12], who found that CI improves postural control through its impact on the auditory system. Therefore, the results of this study may in some way explain the changes in the balance performance after changing the conditions of visual perception, which in turn may be a consequence of the dominance of different compensatory mechanisms’ models. 

Most studies indicated an improvement in postural control after CI surgery [19,20]. However, Louza et al. [13] did not confirm the relationship between neuromuscular control and the participant’s age. This is contrary to the results of our study, which found age as a predictor of the velocity of vCOP (decrease) in the SG group (CI). At this point, it should be emphasized that the impact of age on the postural control of CI students may depend on the participants’ age at CI surgery and the time of adaptation from one somatosensory compensatory model to another, i.e., from the kinesthetic–tactual–auricular (that might be predominant before CI) to the kinesthetic–auricular–tactual compensatory model. It has been shown that the postural control in early CI children can be related to the central compensatory mechanisms responsible for the improved adaptation to the somatosensory compensatory mechanisms by improving the functions of the vestibule of the ear [21]. Thus, it seems that the velocity of vCOP can be affected by both the participant’s age and the age at CI surgery. 

In both groups, regardless of the visual perception, changes in the area were found to be related to the type of hearing device (CI/HA). However, greater changes were observed in the CI group. Therefore, the present study indicated that CI may have affected the neuromuscular control in a different way than HA. To the best of the authors’ knowledge, there is currently no evidence available to be compared with our study. Therefore, to fully understand the complexity of the interactions that occur between neuromuscular control and CI/HA, further studies are needed to evaluate the effects of auditory stimulation (activation/deactivation) on the postural balance in late-CI individuals.

### Limitations

The present study has several limitations that need to be addressed: (1) the study did not include a control group of adolescents without hearing impairments, which would enable comparing the strength and differences in the somatosensory compensatory mechanisms between the CI and HA groups; (2) the study was conducted only on inactive adolescents and thus the findings cannot be generalized to physically active people; (3) we analyzed only the condition of visual perception; therefore, further studies including the condition of auditory stimulation are needed to generalize the results.

## 5. Conclusions

1. Late unilateral CI may impact the activation of different models of the auditory compensatory mechanism than HA, which is related to neuromuscular control.
2. The values of vCOP can be predicted by age in late-CI individuals.
3. Visual perception seems not to be related to the values of the area, which can be impacted both by CI and HA.

## Figures and Tables

**Table 1:** The descriptive statistics of the participants’ characteristics.

Variables	SG Unilateral CI and Unilateral HA (n = 27; nF = 13, nM = 14)		SG Females vs. Males	CG Bilateral HA (n21=; nF11=; nM = 10)		CG Females vs. Males
	Females	Males	*p*-Value	Females	Males	*p*-Value
	Mean ± SD	Mean ± SD		Mean ± SD	Mean ± SD	
Age (years)	17.0 ± 1.9	16.6 ± 1.7	*p* < 0.05	17.5 ± 2.42	16.4 ± 1.8	*p* < 0.05
Age of CI (years)	8.9 ± 3.9	9.2 ± 3.2	*p* < 0.05	N/A	N/A	N/A
BH (cm)	163.3 ± 7.4	175.0 ± 8.7	*p* > 0.05	163.5 ± 6.6	173.1 ± 8.5	*p* > 0.05
BM (kg)	57.6 ± 10.2	61.8 ± 11.8	*p* > 0.05	56.4 ± 11.0	63.2 ± 12.7	*p* > 0.05
BMI	21.6 ± 3.8	20.0 ± 3.1	*p* > 0.05	21.0 ± 3.7	20.9 ± 3.0	*p* > 0.05
FM (%)	26.2 ± 7.4	16.6 ± 6.0	*p* > 0.05	24.8 ± 8.4	19.8 ± 5.3	*p* > 0.05
FM (kg)	15.5 ± 6.6	10.9 ± 5.9	*p* > 0.05	14.8 ± 7.2	13.0 ± 6.4	*p* > 0.05
FFM (kg)	41.6 ± 5.1	50.8 ± 6.9	*p* > 0.05	41.9 ± 4.9	49.4 ± 8.3	*p* > 0.05
TBW (kg)	30.5 ± 3.7	37.7 ± 5.6	*p* > 0.05	31.2 ± 2.7	35.3 ± 4.4	*p* > 0.05
Foot length (cm)	38.6 ± 1.2	43.1 ± 1.5	*p* > 0.05	39.0 ± 2.0	42.7 ± 1.7	*p* > 0.05
Duration of using of the hearing device (CI/HA) (years)	8.4 ± 3.2	9.2 ± 3.2	*p* < 0.05	15.9 ± 2.7	14.8 ± 2.2	*p* < 0.05

SG—study group; CG—control group; CI—cochlear implantation; HA—hearing aid; n—total number of participants; nF—number of females; nM—number of males; SD—standard deviation; N/A—not applicable; BMI—body mass index; FM—fat mass; FFM—fat free mass; TBW—total body water.

## Data Availability

The data used and analyzed during this study are available from the corresponding author upon reasonable request.
